# Evaluation of roots, root canal morphology, and bilateral symmetry of maxillary first molars in a Syrian subpopulation using cone beam computed tomography

**DOI:** 10.1002/cre2.782

**Published:** 2023-09-11

**Authors:** Safaa Allawi, Helen Ayoubi, Mouhammad Al‐Tayyan, Eyad Toutangy, Yasser Alsayed Tolibah

**Affiliations:** ^1^ Department of Endodontics, Faculty of Dentistry Damascus University Damascus Syria; ^2^ Department of Orthodontics Syrian Private University Damascus Syria; ^3^ Department of Pediatric Dentistry, Faculty of Dentistry Damascus University Damascus Syria

**Keywords:** cone beam computed tomography, maxillary first molars morphology, Syrian population, Vertucci classification

## Abstract

**Objective:**

Successful endodontic treatments require a comprehensive knowledge of the root canal anatomy, so this study aimed to investigate the number of roots, configurations of root canals, and their bilateral symmetry of maxillary first molars in the Syrian subpopulation, and also the effect of gender on this symmetry.

**Materials and Methods:**

The study sample consisted of 250 cone beam computed tomography images (140 for females; and 110 for males), including 500 maxillary first molars. Images were investigated by two endodontists. Root number and canal configuration in each root were recorded, according to Vertucci classification, by studying the image at all levels (axial, coronal, sagittal, oplique, and three‐dimensional) to assess the bilateral symmetry and its relation to gender. Statistical analysis was performed with SPSS and the *χ* ^2^ test was used to compare the bilateral symmetry in males and females.

**Result:**

The most common shape of the maxillary first molars was three roots (97.6%). All the roots are symmetrical by 100% in both genders. The root canal configuration was mainly Vertucci type I classification in the distobuccally (73.6%), and palatal root (98%). While the most common types in mesial root were type II (33.3%), this root showed all different types of Vertucci classifications except type VIII, and the proportion of symmetry was (37.2%) without significant difference between the gender (*p* = .441).

**Conclusion:**

Most maxillary first molars in a Syrian population were three‐rooted with four root canals (type II), the numbers of roots achieved perfect symmetry 100%, and higher than symmetry ratios in the number of canals and canals configurations (37.2%).

## 1 INTRODUCTION

The morphology of the roots is complex and variable, and one of the most common causes of endodontic treatment failure is a missed canal because of a lack of knowledge of the root canal system anatomy, especially in the maxillary first molars (Torabinejad et al., 2020; Vertucci, 2005).

A detailed understanding of the maxillary first molars anatomy is essential because it is one of the first permanent teeth to erupt at early dentition, has a high rate of caries infection, and is one of the most treated teeth (Olcay et al., 2018).

Unfortunately, treatment often fails due to neglect of the second mesial buccal canal's (MB2) presence, and this missed untreated canal potentially initiates the disease of apical periodontitis (Khalifa et al., 2023). Hence, studies are required to well‐understand the internal anatomy of the roots to provide the best endodontic treatment (Aminoshariae et al., 2018).

The development of techniques, such as microscopy, can raise the percentage of clinically detecting MB2 by ensuring a good view and color contrast of the pulpal chamber (Khalifa et al., 2023). Likewise, the development of radiography, especially three‐dimensional (3D) imaging, has a crucial role in predicting the number of root canals and their configuration before starting the treatment, and thus good planning for opening the endodontic chamber and preserving dental structure (Boquete‐Castro, 2022).

Although periapical radiography is taken during clinical endodontic treatments, it lacks the third dimension, the buccolingual one. Thus, cone beam computed tomography (CBCT) was developed to solve this problem by showing the third dimension with a more in‐depth understanding of root canal systems, including anatomical abnormalities by image resolution; but some artifacts resulting from the presence of metals, whether crowns or posts, may hinder the clarity of noteworthy details (Soğur et al., 2012).

In addition, the CBCT is noninvasive, less time‐consuming, and has a lower radiation dose than a computed tomography (CT) scan. However, it provides fewer details than micro‐CT images (Nascimento et al., 2019).

Historically, many studies have been performed to understand the internal anatomy of maxillary first molars, including laboratory studies on teeth that have been extracted, or clinical studies (Baratto Filho et al., 2009).

The development of CBCT enables endodontists to evaluate the anatomy of teeth without making interference on a large samples, which can assess the variations in gender, age, ethnicity, and genetic background (Al‐Saedi et al., 2020).

The knowledge of bilateral symmetry when treating opposing teeth in the same patient is a crucial factor, as it enables dentists to predict the presence of additional roots or canals on the opposite side of the already treated tooth, increases the chances of success of endodontic treatments, and reduces failure rates to the least possible (Furuse et al., 2014).

This study is considered unique because it is the first and only study performed in Syria aiming to investigate the number of roots, root canals' morphology of the maxillary first molars, and the bilateral symmetry of the Syrian subpopulation using the CBCT. It also aimed to explore the effect of gender on these morphological aspects. Thus, the results of this study are valid for practicing dentists in predicting the number of roots and root canals before starting endodontic treatment.

## 2 MATERIALS AND METHODS

This retrospective cross‐sectional study was conducted from November 2020 and January 2022 at the Department of Endodontics, Faculty of Dentistry, Damascus University. The study protocol, questionnaires, and informed consent are in full accordance with the ethical guidelines of the Declaration of Helsinki. The Local Research Ethics Committee of the Faculty of Dentistry (UDDS692—10092020/SRC‐3236) approved this research project.

The sample consisted of 766 images, which had been taken from the most recent images at the orthodontics and maxillofacial departments at Damascus University Faculty of Dentistry. Two hundred fifty images of patients, whose ages ranged between 15 and 65 years, fulfilled the selection criteria; their maxillary first molars on the right and left sides were fully erupted and had complete roots with closed apex. The rest of the images were excluded: one of the maxillary first molars was missing or exhibited resorption (internal or external), associated with apical lesions, included post and core, or presented calcified canals, presence of endodontic treatment, and teeth showing malformation. Therefore, the sample size was 250 CBCT images containing 500 maxillary first molars. The sample size was estimated to be a little more than similar studies (Plotino et al., 2013). All X‐rays included in the sample were taken by SCANORA™ 3D 2013 (Soredex). This device provides scanning of all images covering the entire maxillofacial region using the following parameters: scanning field dimensions (FOV) of 145 × 130 mm, 15 MA Intensity, 85 KV Voltage, 0.25 mm Voxel's size (standard resolution mode), and 12 s exposure time.

Two PhD students from the Department of Endodontics performed image evaluation and analyzed data. They were trained to read CBCT images and their radiographic findings were compared. The assessors then reread images a month after the first reading to ensure the accuracy of the recorded results. Then, 10% of the sample was chosen randomly and presented to a third PhD student in the Department of Endodontics. The results of the third endodontist's reading reassured those of the two previous assessors (by Cohen *κ* test; *K* = 0.990, and *p* ≤ .001). The data of each image was processed using the OnDemand3D computer software of CyberMed SCANORA 3D 2013 (Soredex). Settings were set to Maximum Intensity projection, Sharpened, and the thickness of the studied slices was 0.25 mm.

The number of roots, the number of canals, and their configuration were verified by studying the image at all levels: (axial, coronal, sagittal, oplique, and 3D). Orientation was made from the pulpal chamber toward the root apex and from the mesial toward the distal. The images were enlarged and the view settings, such as density, contrast, and invert were changed for better visibility of the anatomical details, which ensured the correct reading of the images. Statistical analysis was performed using SPSS v.26 software (IBM) at the statistical significance level <.05, then a *χ* ^2^ test was used to compare the symmetry of the left and right teeth in males and females. The Vertucci classification was based on eight main classifications which are (Vertucci, 2005):

Type (I): (1‐1) A single canal from the pulp chamber to the apex.

Type (II): (2‐1) Two separate canals leaving the chamber but merging short of the apex to form a single canal.

Type (III): (1‐2‐1) A single canal from a pulp chamber that divides into two, and subsequently merges to exist as one.

Type (IV): (2‐2) Two distinct canals from the pulp chamber to the apex.

Type (V): (1‐2) A single canal leaving the chamber and dividing into two separate canals at the apex.

Type (VI): (2‐1‐2) Two separate canals leaving the pulp chamber, merging in the body of the root, and dividing again into two distinct canals short from the apex.

Type (VII): (1‐2‐1‐2) A single canal that divides, merges, and exits into two distinct canals short from the apex.

Type (VIII): (3‐3) three distinct canals within one root from the pulp chamber to the apex.

Data were collected and recorded on Microsoft Excel, and then statistical tests were carried out using SPSS v.26 (IBM) with a significance level of .05. The *χ* ^2^ test was used to compare the symmetry of the left and right teeth in males and females.

## 3 RESULTS

A total of 250 CBCT images of the Syrian subpopulation aged 15–65 years (140 for females and 110 for males) having (500) maxillary first molars were assessed. The majority of them had three roots (97.6%), followed by four roots (1.6%), two roots (0.8%), and no cases of one root (Table 1).

**Table 1:** Number of roots in maxillary first molars.

Number of roots in maxillary first molars (*n* = 500)	Two roots	Three roots	Four roots	*p* Value
Males 220 (44%)	0 (0.0%)	216 (98.2%)	4 (1.8%)	.195
Females 280 (56%)	4 (1.4%)	272 (97.1%)	4 (1.4%)	
Total 500 (100%)	4 (0.8%)	488 (97.6%)	8 (1.6%)	

The number of three roots was higher in males than females (98.2% and 97.1%, respectively) without any significant difference (*p* = .195).

In the three‐rooted maxillary first molars, the root canal configuration was mainly Vertucci type I classification in the distobuccally 73.6% (67.1% males, 78.8% females), and palatal root 98% (95.4% males, 100% females). Mesiobuccally root showed all different types of Vertucci classifications except type VIII. The most common types were type II (33.3%), type VI (30.7%), type IV (18.8%), and additional type (11%) (Table 2).

**Table 2:** Configuration of the root canal system in the maxillary first molars (*n* = 500).

Number of roots	Root	Type I	Type II	Type III	Type IV	Type V	Type VI	Type VII	Type VIII	Additional
Two roots	B	3 (75%)	1 (25%)	0 (0.0%)	0 (0.0%)	0 (0.0%)	0 (0.0%)	0 (0.0%)	0 (0.0%)	0 (0.0%)
	P	3 (100%)	0 (0.0%)	0 (0.0%)	0 (0.0%)	0 (0.0%)	0 (0.0%)	0 (0.0%)	0 (0.0%)	0 (0.0%)
Three roots	MB	10 (2%)	163 (33.3%)	6 (1.2%)	92 (18.8%)	2 (0.4%)	150 (30.7%)	12 (2.5%)	‐	54 (11%)
	DB	360 (73.6%)	63 (12.9%)	20 (4.1%)	2 (0.4%)	5 (1%)	27 (5.5%)	5 (1%)	1 (0.2%)	6 (1.2%)
	P	479 (98%)	4 (0.8%)	4 (0.8%)	2 (0.4%)	0 (0.0%)	0 (0.0%)	0 (0.0%)	0 (0.0%)	0 (0.0%)
Four roots	MB	8 (100%)	0 (0.0%)	0 (0.0%)	0 (0.0%)	0 (0.0%)	0 (0.0%)	0 (0.0%)	0 (0.0%)	0 (0.0%)
	MP	8 (100%)	0 (0.0%)	0 (0.0%)	0 (0.0%)	0 (0.0%)	0 (0.0%)	0 (0.0%)	0 (0.0%)	0 (0.0%)
	DB	8 (100%)	0 (0.0%)	0 (0.0%)	0 (0.0%)	0 (0.0%)	0 (0.0%)	0 (0.0%)	0 (0.0%)	0 (0.0%)
	DP	8 (100%)	0 (0.0%)	0 (0.0%)	0 (0.0%)	0 (0.0%)	0 (0.0%)	0 (0.0%)	0 (0.0%)	0 (0.0%)

The MB2 had a prevalence of 95.6%, and the type II Vertucci classification was the most common canal configuration (33.3%).

There were no significant differences between males and females regarding the symmetry of the number of roots in maxillary first molars, and the symmetry ratio was 100% for both genders. Considering the symmetry in numbers and configuration of the root canal, there was also no significant statistical difference between genders (*p* = .441). The symmetry proportion of the whole sample was 37.2% (34.5% males, 39.3% females) (Table 3).

**Table 3:** Bilateral symmetry of the number of root and root canals in maxillary first molars (summarized by gender).

Gender	Number of roots		*p*	Number of root canals		*p*
	Symmetrical	Asymmetrical		Symmetrical	Asymmetrical	
Males (*n* = 110)	110 (100%)	0 (0.0%)	_	38 (34.5%)	72 (65.5%)	.441
Females (*n* = 140)	140 (100%)	0 (0.0%)		55 (39.3%)	85 (60.7%)	
Total (*n* = 250)	250 (100%)	0 (0.0%)		93 (37.2%)	157 (62.8%)	

**Figure 1:**
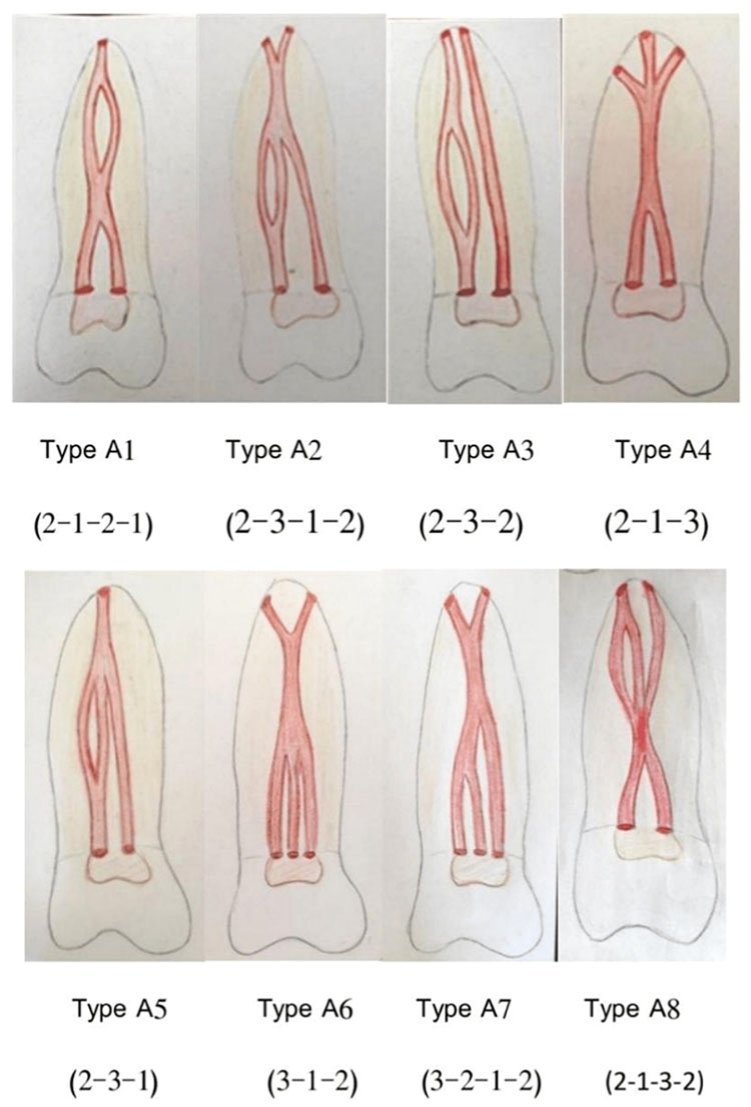
A set of additional modifications found in the current study.

A set of additional modifications we found in our research (Figure 1).

Type A1 (2‐1‐2‐1): Two separate canals leave the pulpal chamber to meet inside the root to form one canal, then return to separate into two canals, and end with one apex.

Type A2 (2‐3‐1‐2): Two separate canals leave the pulpal chamber, then one of them separates into two canals, meets one canal within the root, and separates at the end of the root to end with two different apexes.

Type A3 (2‐3‐2): Two canals leave the pulpal chamber, then one of them separates into two canals, then they return and converge, travel with the other canal, and end with two different apexes.

Type A4 (2‐1‐3): Two canals leave the pulpal chamber, then meet and form one canal in the middle of the root, which separates at the end of the root into three canals, ending in three apexes.

Type A5 (2‐3‐1): Two canals leave the pulp chamber, then one of them separates into two canals, and they meet at the end of the root with one apex.

Type A6 (3‐1‐2): Three canals leave the pulp chamber, then meet in one canal in the middle of the root, and separate into two canals at the end of the root with two different apices.

Type A7 (3‐2‐1‐2): Three canals leave the pulpal chamber, then meet two canals, and also meet one canal, and return to branch into two canals at the end of the root, and end with two different apices.

## 4 DISCUSSION

Root canal treatments may fail, especially in maxillary first molars, as a consequence of missing canals, due to the lack of knowledge of the root canal system anatomy (Cantatore et al., 2006). Hence, such valuable information has a remarkable impact on practice to inspect the possibility of similarities between the previously treated teeth and the bilateral ones within the same jaw. Therefore, clinicians should be aware of the anatomy and plan treatment to better disinfect and obturate the root canal system, especially in the maxillary molar, to achieve optimal endodontic treatment.

The CBCT has been one of the favorite tools for such investigation due to its noninvasive nature, which provides a deeper understanding of the anatomy of root canal systems. However, it also has some limitations, the low resolution can cause artifacts resulting from the presence of the metal and increase the possibility of missing fine details of the canal (De Vos et al., 2009).

This study is the first to provide detailed information on the number of roots, root canal configuration, and bilateral symmetry of maxillary first molars in both genders of the Syrian subpopulation, exhibiting numerous morphological diversity of root canal systems within these teeth. The anatomy of root canals was categorized into eight types according to the Vertucci classification (Vertucci, 2005). This classification was adopted in the current study as a reference because it is the most widely used classification in previous studies (Ghobashy et al., 2017; Plotino et al., 2013).

Therefore, it can easily compare the results of this study with those obtained in previous studies, including gender, ethnicity, age, geographical location, and evaluation methods; in vivo through 2D or 3D radiographic approaches methods or in vitro were teeth rendered transparent by placing them in methyl salicylate, then injected methylene blue dye into them to color entire pulp then to be observed under Dental Operating Microscope at ×12 magnification to identify the morphological complexity of root canals (Sert & Bayirli, 2004).

In maxillary first molars, the most frequently observed root numbers were three (97.6%), followed by four (1.6%), and two  (0.8%), while only one root case was not found. These findings of the three roots align with the range reported by studies conducted on Turkish (97.8%), Iranian (94%), and Saudi (94%) populations (Alrahabi & Zafar, 2015; Ghoncheh et al., 2017; Kalender, 2016). This relative consistency can be attributed to the fact that these countries are located in similar geographical areas. Moreover, they used a similar methodology to determine the molar morphology. However, the proportion of the three roots in the current study was lower than the results of the study conducted in Moscow and Poland, where the prevalence of three roots was 100% (Olczak & Pawlicka, 2017; Razumova et al., 2018).

Molars with four roots and four canals were found at a rate of (1.6%). The results of this study were in agreement with Rouhani's study on the Iranian population (1.6%) (Rouhani et al., 2014) but differed from the results of Kim's study on Korean society, where no molars with four roots were observed (Kim et al., 2012). This difference may be attributed to the disparity in radiographic interpretation, as the thickness of the sections studied in his research was 1 mm while in our study it was 0.2 mm, in addition to the regional population diversity.

Molars with only one root were not found in this study unlike the Neelakantan study in India, which reported the existence of a maxillary first molar with one root and one canal. However, this study was conducted on extracted teeth, and then the first molars were classified based on the shape of the crown. Thus, this difference may be attributed to the difference in the study method between the laboratory study on extracted teeth and the radiological study (Neelakantan et al., 2010).

In the three‐rooted molars, the diversity of root canals in the mesial root was high, with seven types of Vertucci classification seen, but type VIII was not seen in any case. The most common was type II (33.3%), followed by type VI (30.7%), then IV (18.8%), in addition to additional types (11%). These findings align with the results of some studies on the Saudi, Emirati, and Spanish populations where type II was the most common (51.65%, 59%, 56.5%) (Al Mheiri et al., 2020; Mirza et al., 2022; Pérez‐Heredia et al., 2017). The reason for agreement may be the use of the same study method.

The findings of the current study differed from the results of studies conducted in Korea and India, as type IV was most common in the mesial root (Kim et al., 2012; Neelakantan et al., 2010), while type I was the most common in Japan at 37% (Matsunaga et al., 2022) and the type V was the most common in Brazil which use the most accurate micro‐CT (Camargo Dos Santos et al., 2020). These differences may be due to regional population diversity and the genetic predisposition of different communities. Moreover, the study design (radiological or laboratory study) and additional modifications also have a role in these differences, which was consistent with those found in previous studies conducted on different populations and diverse teeth (Al‐Qudah & Awawdeh, 2009; Sert & Bayirli, 2004).

In the current study, the most common type in the distal root was type I (73.6%), while the least common type was an additional type (1.2%), which agreed with the results of Zheng's study on a sample of Chinese society, where the additional type was found in 1.8%. This agreement may be attributed to the similarity in the radiographic interpretation and the thickness of the studied sections (Zheng et al., 2010).

The most common type in the palatal root was type I (98%), followed by types II and III (0.8%) The presence of two canals in the palate root agreed with the Saudi study (0.8%) (Alhujhuj et al., 2022)

Some case reports found three canals in the palatal root (Pan et al., 2019). These higher percentages of root canals reported can be explained by the difference in the study methods used, in addition to the differences in the accuracy of the CBCT devices, voxel size, and the thickness of the slices studied. Studies have shown that the ideal voxel size for recognizing canal configuration is 0.2 mm (Bauman et al., 2011).

The percentage of MB2 canal presence in the maxillary first molars in this study was high (95.6%), and this result agreed with Kalender's study, where the percentage of MB2 presence in the Turkish sample was (93.5%) (Kalender, 2016). It was higher than the percentage in the Saudi sample (85.6%), (Mirza et al., 2022) and also higher than the results of the study conducted on the Brazilian sample (42.63%) (Silva et al., 2014). The reason for this difference may be the genetic mixing in this community, as the origins of grandparents are diverse—a mixture of European, African, and American origins. Additionally, the difference in a voxel size of 200 mm in that study may have also contributed to the variance in the results (Pena et al., 2009; Silva et al., 2014).

The proportion of symmetry for the numbers of roots in this study was 100% for both genders without a significant statistical difference, and this finding agreed with Mashyakhy's study on the Saudi population, where the proportion of root numbers corresponded to 100% (Abu‐Melha, 2021). However, this result was higher than the result of Zhang's study on a sample of Chinese society, as the symmetry rate was 95.2% (Zhang et al., 2017). The reason for the symmetry may be due to the genetic predisposition of the population of these communities. Nevertheless, little research has been done on the ratio of bilateral symmetry of the same patient. Although these studies have reported different symmetry ratios, due to the diversity of countries, they all suggest that symmetry ratios have been always greater than asymmetry ratios (Plotino et al., 2013).

These results have great clinical importance during the treatment of a molar for a patient who had already undergone treatment on the opposite side. The previously treated molar serves as a vital guide for the dentist, expecting to help them anticipate the number of roots and canals and the possibility of the existence of MB2, thus enabling them to provide the best treatment for the patient.

As for the symmetry in the numbers and configuration of root canals in the Syrian subpopulation, there was no significant statistical difference between genders (*p* = .441). The percentage of symmetry in the entire sample was (37.2%). This result differed from Yemi Kim, where the percentage of symmetry root canals in Korean society was (88.10%). The reason for this discrepancy may be due to the focus on the symmetry of the presence or absence of the second mesial canal MB2 without a comparison of the distal and palatal roots, in addition to the difference in voxel size, 0.167 mm, and thus the variance in the radiographic interpretation of the images (Kim et al., 2012).

The main limitation of this study is that images from all Syrian cities were not collected; only images from the Syrian capital “Damascus” were obtained, potentially causing some bias during image collection due to the retrospective design of the study. Additionally, relying on one method in the study without comparing these results with other methods is another limitation that should be acknowledged.

Further studies in different Syrian cities should be performed to assess the relationship between age, gender, and canal configuration, providing a clearer and more comprehensive understanding of the canals of the maxillary first molars of Syrian society. Another area for further research involves using a larger sample size and using more accurate imaging methods such as CT scan.

## 5 CONCLUSIONS

Within the limitations of this study, it can be concluded that the most common anatomical form of the maxillary first molars in the Syrian subpopulation was three separated roots with four canals. The numbers of roots achieved perfect symmetry 100% and higher than symmetry ratios in the number of canals and canals configurations (37.2%) without significant statistical difference between the gender.

## AUTHOR CONTRIBUTIONS

Safaa Allawi and Eyad Toutangy conceptualized the idea, read the CBCT images, and contributed to the writing and documentation. Helen Ayoubi and Mouhammad Al‐Tayyan conceptualized the idea and supervised the MSc's thesis for Safaa Allawi. Yasser Alsayed Tolibah contributed to the interpretation of data, revision, formatting, and reediting of the manuscript. All authors read and agreed to the published version of the manuscript.

## CONFLICT OF INTEREST STATEMENT

The authors declare no conflict of interest.

## ETHICS STATEMENT

The study was conducted according to the guidelines of the Declaration of Helsinki and approved by the Local Research Ethics Committee of the Faculty of Dentistry, Damascus University (UDDS692—10092020/SRC‐3236). Informed consent was obtained from all subjects/caregivers involved in the study.

## Data Availability

Data are available upon written request to the corresponding author.
